# Supplementary material for: Standardized approach to the conservative surgery of hepatic cystic echinococcosis: A prospective study
Source: PLoS Negl Trop Dis. 2024 Jun 26;18(6):e0012289. doi: 10.1371/journal.pntd.0012289 (PMC11232992; doi:10.1371/journal.pntd.0012289)
Supplement: Attachment — Submitted filename: Review (6).docx [file pntd.0012289.s001.docx]

This study evaluated the results of a standardized approach of conservative surgery for hepatic cystic echinococcosis (HCE) in 50 patients (106 cysts). It was found that only two patients experienced a deep surgical site infection in the form of an external bile leak postoperatively, which resolved with conservative management. Additionally, no cases of recurrence were reported during a median follow-up of 24 months.

The study is well written and well-structured, and the quality of the English language is good. The novelty and strength of the paper is the detailed description of the surgical method, especially how many cystobiliary fistulas were observed intraoperatively and how they were dealt with.

But some points are to be addressed:

1. The introduction briefly mentions previous studies for radical and conservative surgical management of HCE. However, many sentences in this section have no references.

2. The authors should use the term “conservative surgery” instead of “conservative treatment”. “Conservative treatments are medical therapy or PAIR method.

3. Authors state that the study is a prospective study, and a comparative study was done to compare this cohort to another population operated on using the usual technique before standardization. What are the results of the comparative study? Have they already been published? Not reported and not published results cannot be used as comparative group or for population/power calculations. This is a major flaw and must be addressed.

4. Row 142: „a 10 mm trocar without he mandrel…“ did authors mean “the mandrel”?

5. Row 147: „The residual cavity is washed with hypertonic saline solution in the absence of any obvious large cystobiliary fistula”. Many authors believe that using hypertonic saline solution might lead to cholangiocellular damage and ischemic cholangiopathy if small invisible bile ducts exist in the cyst cavity. Did authors have such complications. Could they discuss this in the text.

6. There is no data about Statistical analysis. With which software the analysis has been done? Which statistical tests were administered?

7. The first paragraph of the discussion is a repetition of introduction and can be deleted. I suggest that the authors report a brief summary of the main results instead in the first paragraph.

8. The reported rate of bile leak in this study is 1.9%, which is lower than similar studies. Authors discuss that „the variability in the reported rates of complications could be attributed to the different definitions of external biliary fistula used by various authors “. I agree to this fact, and I agree that the using of a standardized procedure and bile leak tests (methylene blue) are some reasons explaining the good results. But another important reason is that the patient’s cohort includes selected cases. As mentioned in methods: „we excluded patients who had cysts complicated with a large cystobiliary fistula revealed by acute cholangitis or acute pancreatitis or presence of cystic debris in the common bile duct on preoperative imaging “. This fact should be further explained. How many patients were excluded and what was the performed procedure in these patients.

9. The content of the rows 387-390 is the same as 381-386. One of the paragraphs should be deleted.

10. The most recent study published in conservative surgery from Abbasi et al (doi: 10.1371/journal.pntd.0011724) showed that a risk factor of postoperative bile leak is the cyst diameter. Can you discuss this fact. Did you observe more cystobiliary fistulas intraoperatively in patients with larger cysts or was the cyst diameter related to cystobiliray fistulas?

11. How long was the hospital stay of the patients with bile leaks and what kind of conservative management was exactly performed? (e.g., watch and wait, antibiotic therapy, endoscopic interventions etc.)

In conclusion, I suggest major revisions.
