# Supplementary material for: Standardized approach to the conservative surgery of hepatic cystic echinococcosis: A prospective study
Source: PLoS Negl Trop Dis. 2024 Jun 26;18(6):e0012289. doi: 10.1371/journal.pntd.0012289 (PMC11232992; doi:10.1371/journal.pntd.0012289)
Supplement: Attachment — Submitted filename: Review (6).docx [file pntd.0012289.s002.docx]

This study evaluated the results of a standardized approach of conservative surgery for hepatic cystic echinococcosis (HCE) in 50 patients (106 cysts). It was found that only two patients experienced a deep surgical site infection in the form of an external bile leak postoperatively, which resolved with conservative management. Additionally, no cases of recurrence were reported during a median follow-up of 24 months.

The study is well written and well-structured, and the quality of the English language is good. The novelty and strength of the paper is the detailed description of the surgical method, especially how many cystobiliary fistulas were observed intraoperatively and how they were dealt with.

But some points are to be addressed:

**1.The introduction briefly mentions previous studies for radical and conservative surgical management of HCE. However, many sentences in this section have no references.**

We added the relevant references: (2,3,4,5).

2. The authors should use the term “conservative surgery” instead of “conservative treatment”. “Conservative treatments are medical therapy or PAIR method.

We changed the ambiguous term “conservative management” to “conservative surgery”.

3. Authors state that the study is a prospective study, and a comparative study was done to compare this cohort to another population operated on using the usual technique before standardization. What are the results of the comparative study? Have they already been published? Not reported and not published results cannot be used as comparative group or for population/power calculations. This is a major flaw and must be addressed.

This was mistake from our part. We did not undertake any comparative study. The aim of our study was to achieve surgical outcomes of conservative surgery comparable to radical surgery.

4. Row 142: „a 10 mm trocar without he mandrel…“ did authors mean “the mandrel”?

“the mandrel” was corrected.

5. Row 147: „The residual cavity is washed with hypertonic saline solution in the absence of any obvious large cystobiliary fistula”. Many authors believe that using hypertonic saline solution might lead to cholangiocellular damage and ischemic cholangiopathy if small invisible bile ducts exist in the cyst cavity. Did authors have such complications. Could they discuss this in the text.

Sclerosing cholangitis as a result of using it as a scolicidal solution is a major concern to us when we perform the sterilization of the cyst cavity. Hypertonic saline wash is used only after we have ensured that any opening the bile ducts has been closed. In the case of large CBF or any any overt cystobiliary fistula, normal saline will be used instead. We clarified how this was avoided in our study: *“Once the intracystic pressure decreases and cyst becomes flaccid, a 2 cm incision is carefully performed on the pericyst. The cyst content is fully aspirated, and the cyst cavity is generously irrigated with normal saline, aspirating and replenishing the solution 2 to 3 times to thoroughly dilute any remaining protoscoleces. The cavity is then swabbed with gauze dipped in 20% hypertonic saline solution and it the swab is left in the cavity for 10 minutes. Direct washing with hypertonic saline solution is not done to prevent sclerosing cholangitis.*

*The cyst incision is widened to better asses the cavity and a visual exploration of the residual cavity is carried out to detect any bile leakage or visible cystobiliary fistula. The inner wall is gently scraped using a curette to remove membranous debris. Any visible CBFs detected during the visual exploration of the cavity are sutured using Vicryl® 2/0.*

*After the leakage tests are negative or in the absence of any CBFs, the cavity is thoroughly washed with hypertonic saline. The protruding part of the pericyst is then resected, preventing any live protoscoleces from leaking outside the cavity”.*

6. There is no data about Statistical analysis. With which software the analysis has been done? Which statistical tests were administered?

We added a statistical analysis section in “Patient and methods” to detail the statistical methods used.
*“IBM SPSS Statistics for Windows, version 26 (IBM Corp., Armonk, N.Y., USA) was used for data entry and for all statistical analyses. Frequencies were calculated for qualitative variables, while the median (semi-interquartile range (SIR)) was computed for quantitative variables. For overall morbidity and nonspecific morbidity, the analysis was done per patient. For specific complications and cyst recurrence, the unit of study was the cyst. Therefore, for patients with two or more cysts, each cyst was considered independent.”*

7. The first paragraph of the discussion is a repetition of introduction and can be deleted. I suggest that the authors report a brief summary of the main results instead in the first paragraph.

We deleted the first paragraph and added a summary of our results.

8. The reported rate of bile leak in this study is 1.9%, which is lower than similar studies. Authors discuss that „the variability in the reported rates of complications could be attributed to the different definitions of external biliary fistula used by various authors “. I agree to this fact, and I agree that the using of a standardized procedure and bile leak tests (methylene blue) are some reasons explaining the good results. But another important reason is that the patient’s cohort includes selected cases. As mentioned in methods: „we excluded patients who had cysts complicated with a large cystobiliary fistula revealed by acute cholangitis or acute pancreatitis or presence of cystic debris in the common bile duct on preoperative imaging “. This fact should be further explained. How many patients were excluded and what was the performed procedure in these patients.

Patients with large CBFs had to undergo other techniques to manage the fistulas since simple suturing within the pericyst is not very effective. Peripheral cysts need to be treated with radical methods but are however not feasible for central cysts with close proximity to major veins or biliary structures. This often entails complex liver surgery. Therefore, other techniques such as internal trans-fistulary drainage or cystobiliary disconnection (Perdromo technique) needs to be used. Moreover, in patients with acute cholangitis and pancreatitis, the standardized technique is not ideal for these patients since operative times could be longer. Many studies, will have selection biases, with them including both patient with large fistulas and occult fistulas. Including them, will make our cohort of patients heterogeneous.

We added the paragraph: *“We distinguish 2 types of cysts: cysts with overt, large CBF and cysts presenting occult fistulas. Management differs for these 2 types. For large and peripheral* *cysts, radical resection is preferred. However, cysts with close proximity to major veins and biliary structures, internal trans-fistulary drainage or cystobiliary disconnection (Perdromo technique) is performed since simple suturing of the fistula within the pericyst is not effective. Thus, only patients with occult fistulas were included, to get a homogeneous population and minimize selection bias.”*

9. The content of the rows 387-390 is the same as 381-386. One of the paragraphs should be deleted.

The redundant facts were removed.

10. The most recent study published in conservative surgery from Abbasi et al (doi: 10.1371/journal.pntd.0011724) showed that a risk factor of postoperative bile leak is the cyst diameter. Can you discuss this fact. Did you observe more cystobiliary fistulas intraoperatively in patients with larger cysts or was the cyst diameter related to cystobiliray fistulas?

Cyst size is indeed a predictive factor of cystobiliary Fistulas. This was demonstrated by the study by Abbasi et al and Trigui et al. with both citing that larger cysts have more cystobiliary fistulas. This was also found in our study and added it to our results and discussed these finding in the discussion part. *“We also found that cysts CBFs were larger (mean diameters of 6.1± 0.6 cm without CBFs vs 8.0 ± 0.5 cm with CBFs), p = 0.024.”*

11. How long was the hospital stay of the patients with bile leaks and what kind of conservative management was exactly performed? (e.g., watch and wait, antibiotic therapy, endoscopic interventions etc.)

We added details about the management of these patients. *“The patients were started on antibiotic course of intravenous cefotaxime and metronidazole upon detection of the fistula. The average output of the fistula was 106 ml/day and it stopped being productive after a mean duration of 25 days. No need for sphincterotomy or surgery was required for these patients. Both patients had a hospital stay of 14 days. No patients developed injury to bile ducts as a result of hypertonic saline use during the sterilization of the cyst.”*

Reviewer 2

1. Please get help of someone fluent in English. Many grammar errors are present.

2. Abstract:

1-patients:

•	please add duration of study, multicenter study, stage of hydatid cyst and operative approach either open or laparoscope

•	uncomplicated HCE then in exclusion criteria you used ----We excluded patients who had cysts complicated

2-results

•	standardized techniques----please clarify

•	86%---no percentage without number of patients and vice versa. please strict to this information throughout the manuscript

•	the cyst had a median size of 7.4 cm.---- please add minimum and maximum size

•	57 cysts-----please add percentage

•	cystobiliary fistulas-----please differentiate between overt and occult hepatobiliary communication

•	bile leak-----grade of biliary leak

3. Keywords: please add keywords

We added the missing parts to the abstract and added the keywords at the end of the abstract section.

4. Introduction:

•	Hepatic Cystic Echinococcosis of the Liver (HCE)---- revise

We corrected this term.

•	There are two main surgical approaches to treat HCE: conservative treatment and radical treatment ---add reference

We added the reference: Dziri C, Haouet K, Fingerhut A. Treatment of hydatid cyst of the liver: where is the evidence?. World journal of surgery. 2004 Aug;28:731-6.

•	resection of the protruding dome (RPD) as described by Lagrot in 1957--- I think it is more suitable to use term partial cystectomy and omentoplasty. very old reference, please insert a more recent one

We updated these references to a more recent one (3,4). We agree with you, the correct terminology to be used for “resection of the protruding dome” is partial cystectomy. (Vuitton DA, McManus DP, Rogan MT, Romig T, Gottstein B, Naidich A, et al. International consensus on terminology to be used in the field of echinococcoses. Parasite. 2020;27:41.)

•	As opposed to the latter technique, radical management which entails liver parenchymal resections with a non-negligible bleeding risk---please add reference

Several studies have suggested that radical surgery may pose a risk of bleeding due to parenchymal resection. (references 2 and 5)

•	references (3,4)---- reference 3 and 4 did not exclude complicated cysts

References 5 and 6 are correct ones.

•	Several technical refinements----references

We added the relevant references to the technical refinements performed.

“*such as* *liver mobilization, intraoperative ultrasound* *(7), systematic methylene blue injection to detect cystobiliary fistulas**(8) and omentoplasty**(9) have been added to the traditional technique to improve postoperative outcomes.*”

5. patients and methods:

•	Please add a clear paragraph of inclusion and exclusion criteria. I think adding a flow chart of inclusion and exclusion criteria will be helpful.

We added a detailed flowchart of the included and excluded cysts (Figure 1)

*“We included in our study all patients who were operated for HCE in an emergent or elective setting by laparotomy. We excluded patients who had cysts complicated with a large cystobiliary fistula revealed by acute cholangitis or acute pancreatitis or presence of cyst debris in the common bile duct on preoperative imaging which could manifest itself through a common bile duct dilation or direct visualization of obstacle in the common bile duct.”*

•	AF---please define

This was redundant and we removed it.

•	Surgical procedure for the standardized technique--- please add a reference to the techniques used and then describe in short

We added the references 7, 8 and 9.

•	In case of CE1 and CE3a detected on intraoperative ultrasound----what about other stage of the cyst?
We added the CE4 and CE3b type in the following sentence pertaining to mastic contents

•	he---- revise

Corrected to “the”

•	Injection of the dye is performed under low pressure without clamping of the common bile duct. These CBF are 157 sutured using Vicryl® 2/0. Clamping of the main bile duct while injecting the dye ---revise

We rephrased the sentence for clarity. *“Then, clamping the main bile duct during dye injection enables the identification of additional CBF and serves as a leakage test for the previously sutured fistula. The sutures that showed leakage of methylene blue are reinforced, and as well as any additional occult cystobiliary fistulas detected during this methylene blue test. The integrity of the sutures is checked by performing a methylene blue test with and without manual clamping of the common bile duct.”*

•	affixed to----how did you fixed it.

The omentum was placed in the cavity and held in place using Vicryl suture. We corrected the sentence.

•	passive drainage of the residual cavity----site of drain insertion

The drain was inserted in the residual cavity through a stab incision in the right flank.

•  This study was conducted following the Helsinki Declaration—please add

added

**•	what is the protocol of albendazole therapy in your hospitals---please add**

We added dosage and duration of treatment

*“As part of the protocol of study, food was allowed on post-operative day 1 with early mobilization and administration of oral albendazole, 400 mg twice daily. The drain was usually removed on post-operative day 5 before discharge unless a complication is detected.”*

•	please state if there are any cases lost to follow up

No cases were lost to follow up and added this information in the figure.

•	please add statistical analysis paragraph

We added a paragraph about the statistical analyses performed.

•	please add a paragraph about Definitions of outcomes and measurements to include

POBF was defined as a bilirubin concentration in the drain fluid at least three times higher than the serum bilirubin concentration on or after postoperative day three[reference]. Hydatid recurrence was defined as the appearance of new active cysts after surgery[reference]. Postoperative residual fluid cavities were not classified as recurrent HCD[reference]. Postoperative morbidity was assessed using the Clavien-Dindo classification[reference]. Hydatid cyst fluid bilirubin and ALP levels were measured using an automatic biochemical analyzer (AU-400, Olympus)[reference]. All hospitals used the same instrument to measure bilirubin and ALP levels in the cyst fluid. All the machines were calibrated at each institution to ensure consistent reporting. The time from surgery to disease relapse at any site was described as recurrence-free survival.

We improved the paragraph to include the outcomes measured and their definitions.

## *“Definition of outcomes of the study*

*The main complication of conservative surgery of HCE which are:*

- *External Biliary Fistula: We considered any clear bile drainage from the abdominal drain, regardless of the timing, duration, or quantity, as a postoperative biliary fistula (10).*
- *Cavity infection: Defined as the occurrence of an infection in the residual cavity with or without the presence of drainage.*

*These aforementioned complications were grouped under the term "Deep Surgical Site Infection" (DSSI) (11). Cavity infections result from bile accumulation by means of a CBF. External bile leakage shares the same cause. Therefore, the rationale behind grouping these complications together is that they both stem from the common cause. The primary outcome of our study was the occurrence of DSSI in the population which was main specific complication of the conservative surgery. Postoperative morbidity was assessed using the Clavien-Dindo classification (12).*

*The secondary outcomes were the overall morbidity, mortality and disease recurrence. Morbidity was defined as the occurrence of one or more complications during hospitalization or within the 30 days following the surgery and mortality was defined as is defined as death occurring within 30 days following the surgery or during the same hospitalization, regardless of its duration. Recurrent hepatic hydatid disease refers to the emergence of new active cysts following surgery.Top of Form*”

6. Results:

Table 1:

•	Recurrence--- what recurrence do you mean?

We imply the existence of a recurrent cyst at admission and that the patients have been previously treated for HCE. Recurrence of the cystic echinococcosis is fairly common in endemic areas of CE.

•	Recurrence of PHE--- recurrence of pulmonary hydatid cyst?

Yes, we changed the abbreviation in the table caption at the end of the table.

•	Cytolysis—clarify

We replaced the less known term of “cytolysis” with “elevated AST and ALT”

•	Hyperbilirubinemia--- please add bilirubin level

We used a bilirubin threshold of 25 μmol/l

Table 2:

o	unique--- change to solitary

•	WHO Classification--- change to please use WHO-IWGE classification

We changed these.

7. Discussion:

o	need to be rewrite in a better way with starting paragraph about the aim of the study and important points in your study. Furthermore, please strict to your outcomes as I can see that the discussion is so long and many paragraphs are not needed as for example---In most hepatobiliary surgeries, the right subcostal approach was classically the most frequently performed. It allows for liver exposure and access to the elements of the hepatic pedicle. However, it may be less suitable for accessing the hepatic dome. The Makuuchi J-incision provides a better view of the posterior and 341 superior segments (27,28). This approach has the advantage of preserving the vascularization of the abdominal wall and causing fewer complications such as wall abscesses (29). This parameter seems to be correlated with liver mobilization, as it is the cysts located in the upper part of the liver (the dome) that require detachment. To access these cysts more easily, the Makuuchi incision is often

preferred.

We edited the discussion.

o	This could be explained by several mechanisms, such as the negative pressure exerted by the diaphragm, which would reverse the bile flow, or having a suspended non-declining cavity---please add a reference

We added the required reference (40).
